# Effectiveness of Phenolic Compounds against Citrus Green Mould

**DOI:** 10.3390/molecules190812500

**Published:** 2014-08-18

**Authors:** Simona M. Sanzani, Leonardo Schena, Antonio Ippolito

**Affiliations:** 1 Dipartimento di Scienze del Suolo, della Pianta e degli Alimenti, Università degli Studi Aldo Moro, Via G. Amendola 165/A, Bari 70126, Italy; 2 Dipartimento di Agraria, Università degli Studi Mediterranea, Località Feo di Vito, Reggio Calabria 89124, Italy

**Keywords:** oranges, phenolic compounds, postharvest, *Penicillium digitatum*, quercetin, scopoletin, scoparone

## Abstract

Stored citrus fruit suffer huge losses because of the development of green mould caused by *Penicillium digitatum*. Usually synthetic fungicides are employed to control this disease, but their use is facing some obstacles, such public concern about possible adverse effects on human and environmental health and the development of resistant pathogen populations. In the present study quercetin, scopoletin and scoparone—phenolic compounds present in several agricultural commodities and associated with response to stresses—were firstly tested *in vitro* against *P. digitatum* and then applied *in vivo* on oranges cv. Navelina. Fruits were wound-treated (100 µg), pathogen-inoculated, stored and surveyed for disease incidence and severity. Although only a minor (≤13%) control effect on *P. digitatum* growth was recorded *in vitro*, the *in vivo* trial results were encouraging. In fact, on phenolic-treated oranges, symptoms appeared at 6 days post-inoculation (DPI), *i.e.*, with a 2 day-delay as compared to the untreated control. Moreover, at 8 DPI, quercetin, scopoletin, and scoparone significantly reduced disease incidence and severity by 69%–40% and 85%–70%, respectively, as compared to the control. At 14 DPI, scoparone was the most active molecule. Based on the results, these compounds might represent an interesting alternative to synthetic fungicides.

## 1. Introduction

*Penicillium digitatum* [Pers.: Fr.] Sacc. is the causal agent of green mould, one of the most common postharvest diseases of citrus fruit. This wound-obligate pathogen has a relatively short disease cycle (3 to 5 days at 25 °C) and, on a single fruit, can produce 1 to 2 billion conidia that efficiently disperse through the air [1]. *P. digitatum* may attack the fruit on the tree, in the packinghouse, in transit, in storage and in the market. However, handling and storage under ambient conditions particularly favours its growth. During this stage, green mould reaches 60%–80% of decay caused by *Penicillium* genera [2]. Youssef *et al.* [3] evaluated the presence and abundance of *Penicillium* spp. conidia in packinghouses, reporting significantly higher values in “bin emptying” area, with a density exceeding 400 CFU/g fw on fruit surface and 66 CFU on semi-selective PDA plates left open in the atmosphere for 10 min. Moreover, the incidence of penicillium rots showed an increasing trend, with values ranging from 23% (bin emptying) to 40% (calibration).

When permitted, synthetic fungicides are the primary means to control green mould. However, the public growing concern for consequences on human and environmental health of toxic residues [4] and the development of fungicide-resistant strains in pathogen populations [1] have motivated the search for alternative approaches.

Among unconventional control strategies, the induction of fruit resistance, the use of plant or animal-derived products with fungicidal activity and the application of antagonistic microorganisms or physical means can be considered, either alone or as part of an integrated pest management policy [5]. Within plant product category, the role of phenolic compounds in the active expression of resistance has been reported [6]. Some of them occur constitutively in the plant (phytoanticipins), whereas others form in response to biotic or abiotic stresses (phytoalexins), such as injuries [7,8].

For example, the exposure of citrus fruit to salt application [9], heat [10], gamma radiation [11] or ultraviolet (UV) light [12] induced the accumulation in the fruit peel of compounds, as the coumarins scopoletin and scoparone, associated with the development of resistance against fungal pathogens. Moreover, following pathogen infection, tissues of *Morinda tomentosa* Roth. and *Cassia fistula* L. proved to contain several flavonoids including quercetin [13]. Similarly, Mayr *et al.* [14] reported that quercetin glycosides are released from apple cell vacuoles as aglycones, developing their toxic activity after pathogen attack. In a previous study [15], we tested the efficacy of several phenolic compounds including quercetin, scopoletin and scoparone against *Penicillium expansum*, causal agent of the blue mould of apple, and the production of its mycotoxin patulin.

The selective accumulation of quercetin, scopoletin and scoparone in plants, as well as their antifungal character and antioxidant properties [15,16,17,18], make them good “natural pesticide” candidates to improve plant resistance to fungal infections. Although several investigations on the accumulation of phenolic compounds following the induction of host resistance were carried out [7,12,19], to the best of our knowledge, there are no bibliographic records on their direct exogenous application on citrus to maintain their postharvest quality.

The aim of the present investigation was to evaluate *in vivo* the activity of the phenolic compounds quercetin, scopoletin and scoparone against green mould on “Navelina” oranges. Moreover, the putative control effect on *P. digitatum* growth was tested by *in vitro* trials.

## 2. Results and Discussion

The effect of different concentrations of quercetin, scopoletin, and scoparone on *in vitro* radial growth of *P. digitatum* is shown in Table 1. At 3 days post inoculation (DPI), only scoparone at the highest tested concentration (100 µg/mL, 1000 µg/plate), significantly, although slightly (up to 13%) reduced fungal growth. On the contrary, a significant enhancement of *P. digitatum* growth was observed in presence of quercetin at the same concentration. When colony diameters were measured at 6 DPI, all treatments proved to reduce significantly fungal growth at 100 µg/mL, being quercetin the best one with a 14% reduction. These findings are in agreement with previous experiments [15] in which quercetin only slightly (13%) reduced *in vitro P. expansum* growth. On the contrary, scopoletin and scoparone did not have any significant effect. Since quercetin is associated to apples, target host of *P. expansum*, and scopoletin/scoparone to citrus, target host of *P. digitatum*, these results seem to confirm the existence of a specificity in the host-pathogen interaction, as reported by Sanzani *et al.* [20]. Afek and Sztejnberg [21] already proved the *in vitro* activity of scoparone against *P. digitatum*. The ED_50_ for spore germination was 64 µg/mL, whereas, as far as we know, no data on radial growth are available in literature. Similarly, Garcia *et al.* [22] reported the *in vitro* fungitoxic effect of 2 mM scopoletin on germ tube elongation and conidia germination of *Microcyclus ulei*. The better results obtained at a higher concentration seem to suggest that the efficacy of phenolics is dose-dependent, thus, in the future higher concentrations will be tested.

**Table 1:** Effect of quercetin, scopoletin and scoparone at 10 and 100 µg/mL (100 and 1000 µg/plate, respectively) on *Penicillium digitatum* colony diameter (mm) after 3 and 6 days post-inoculation (DPI) at 24 °C in the dark.

Treatment	Colony Diameter (mm)	
	3 DPI	6 DPI
Control [no phenolics]	14.7 ± 1.4 ^b^^,c^	54.2 ± 0.5 ^a^^,b^
Quercetin 10 µg/mL	16.3 ± 0.6 ^a^^,b^	56.5 ± 0.4 ^a^
Quercetin 100 µg/mL	17.8 ± 0.2 ^a^	47.7 ± 1.2 ^d^
Scopoletin 10 µg/mL	14.0 ± 0.4 ^c^^,d^	52.5 ± 1.1 ^b^^,c^
Scopoletin 100 µg/mL	13.0 ± 0.8 ^c^^,d^	51.5 ± 1.8 ^c^
Scoparone 10 µg/mL	14.7 ± 0.5 ^b^^,c^	54.7 ± 1.2 ^a^^,b^
Scoparone 100 µg/mL	12.8 ± 0.6 ^d^	50.3 ± 0.9 ^c^

Each value corresponds to the mean of three replicates ± standard error of the mean (SEM). For each assessment time, values with the same letter are not significantly different according to Duncan’s Multiple Range Test (DMRT, *p* ≤ 0.05).

**Figure 1:**
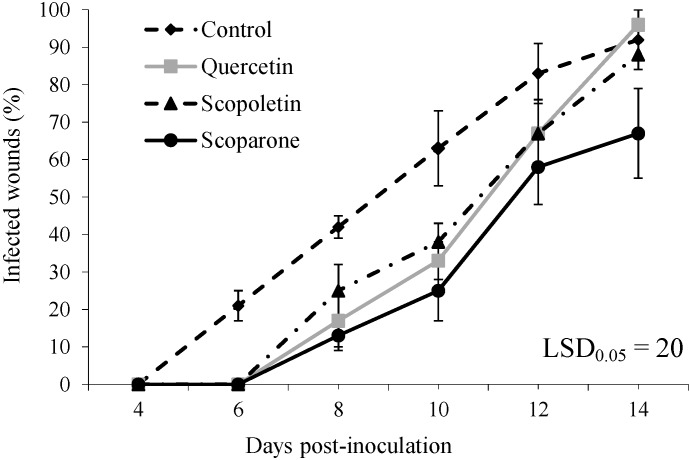
Incidence of decay (infected wounds, %) on “Navelina” oranges treated with quercetin, scopoletin or scoparone (100 µg/wound), inoculated with *Penicillium digitatum*, and incubated at 24 ± 1 °C for 14 days. Untreated fruits served as a control. Each value corresponds to the mean of three replicates ± standard error of the mean (SEM). Means separation according to Fisher’s Least Significant Difference (LSD).

**Figure 2:**
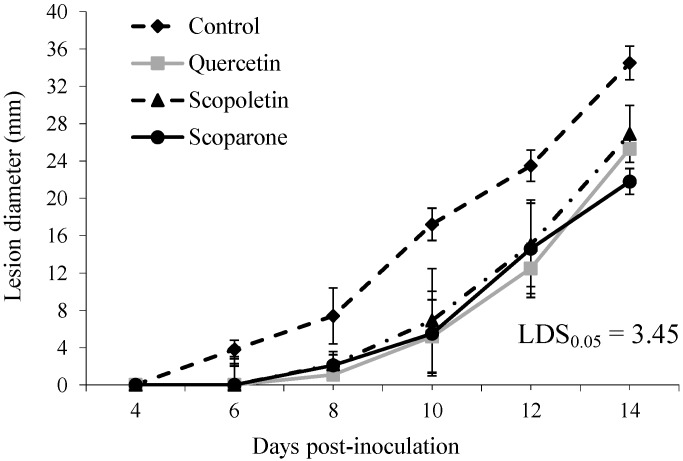
Disease severity (lesion diameter, mm) on “Navelina” oranges treated with quercetin, scopoletin or scoparone (100 µg/wound), inoculated with *Penicillium digitatum* and incubated at 24 ± 1 °C for 14 days. Untreated fruits served as a control. Each value corresponds to the mean of three replicates ± standard error of the mean (SEM). Means separation according to Fisher’s Least Significant Difference (LSD).

Considering the absence of a relevant effect on fungal growth and the results of previous investigations [15], we decided to test quercetin, scopoletin and scoparone at 100 µg/wound against green mould incidence and severity on “Navelina” oranges. Results are reported in Figure 1 and Figure 2, respectively. Concerning incidence of decay, infections started at 6 DPI, *i.e.*, with a 2 day-delay on treated oranges, as compared to the control, and quercetin, scopoletin and scoparone significantly (*p* ≤ 0.05) reduced them by 60%, 40% and 69%, respectively, at 8 DPI (Figure 1). Quercetin and scopoletin maintain their significant effect up to 10 DPI, whereas scoparone was effective for all the incubation period, being the best treatment at 14 DPI (27% reduction). The control activity was confirmed also as far as disease severity concerns (Figure 2). Indeed, *Penicillium* lesion diameters were significantly (*p* ≤ 0.05) reduced by all treatments up to the end of the incubation period. In particular, the three tested phenolic compounds equally reduced disease severity up to 12 DPI (36%–47%), whereas, at 14 DPI, scoparone proved to be the most effective treatment (37% reduction). These results seem to suggest that tested phenolics, rather than completely blocking infections, exert a fungistatic effect. Moreover, the significant efficacy demonstrated by scoparone throughout the incubation period is particularly interesting, considering the average shelf life of oranges in markets and supermarkets.

The role of phenolic compounds in defense mechanisms against pathogens is well known. In particular, quercetin efficacy in reducing apple blue mould incidence and severity has already been reported [15], and results were similar to those recorded in the present study on oranges cv. Navelina. Scopoletin and scoparone induction as alternative control means against blue and green mould of oranges has been also extensively studied [12,23,24,25], however, to date there are no reports on their exogenous application on oranges. Tested phenolics seem to be more effective *in vivo* than *in vitro*. This behavior might be a consequence of: (i) the activation of orange defensive genes; (ii) the addition to phenolics already present in the host, thus reaching a concentration toxic to the fungus; (iii) the lack of detoxification mechanisms in the pathogen for the unknown added compounds; (iv) the interaction with one or more of the pre-existing compounds in fruit tissues, thus forming a new toxic compound.

As well known, during ripening the phenolic profile of fruit skin changes markedly, with a consistent content reduction in mature fruit [26], which becomes at the same time more susceptible to infections. Considering that the inner white layer of citrus peel (albedo) produces quercetin, scoparone, and scopoletin at concentrations even of 80–140 µg/g DW [9,27], it could be conceivable that, with the addition of the tested compounds, a phenolic concentration toxic to the pathogen was restored.

The ability of quercetin to induce resistance to *P. expansum* in apples has been demonstrated [28]. Furthermore, it proved to reduce pathogen toxigenic ability by acting on the biosynthetic pathway of patulin [29], which has not only a health significance, but also was recently classified as a pathogenicity/virulence factor [30].

*P. digitatum* usually needs a wound to initiate the infection process. Harvest and postharvest fruit handling frequently produces wounds on fruit, especially when the peduncle is present. In addition, the environment in the degreening room (warm temperature and high humidity) is ideal for the proliferation of microorganisms and the ineffective sanitizing of packinghouse dump tanks, flumes and hydrocoolers might further promote infection by pathogens. Thus, careful handling of fruits and hygiene of the packing line are of crucial importance in preventing rot development. For instance, water should be treated (either chemically or physically) to prevent unintentional contamination of clean produce [31]. In this context, the use of alternative compounds, such as quercetin, scopoletin or scoparone, might be an interesting alternative to the chemicals currently used, such as chlorine. In fact, our substances showed a promising activity on “Navelina” oranges at an inoculum concentration (5 × 10^4^ conidia/mL) superior to the one commonly present in the floating water (10^3^ conidia/mL) of numerous packinghouses [32]. Moreover, in recent years plant-derived compounds have grown in popularity among consumers [33], especially for their presumed anti-inflammatory and antioxidant properties [34], although toxicity of most plant products for their use as food components has not been proved yet.

Finally, quercetin was successfully applied on apples by dipping in large-scale experiments at a concentration (1.25 g/L) comparable to those of thiabendazole (0.5–1.15 g/L) and imazalil (2 g/L), two fungicides used as postharvest antifungal treatments [1]. Therefore, based on a survey on costs, the application of phenolics might be comparable to the most common fungicides used in packinghouses [15].

## 3. Experimental Section

### 3.1. Chemicals

Sodium dihydrogen phosphate, sodium hydrogen diphosphate, sodium hydroxide, quercetin (3,3',4',5,7-pentahydroxyflavone), scopoletin (7-hydroxy-5-methoxycoumarin) and scoparone (6,7-dimethoxycoumarin) with a purity of >95% were purchased from Sigma-Aldrich (Milan, Italy).

### 3.2. Preparation of Phenolic Compounds Solutions

Stock solutions of quercetin, scopoletin and scoparone were prepared at concentration 10 mg/mL by dissolving pure standards into a mixture of phosphate buffer (50.0 mmol/L, pH 7.4)/NaOH (1.0 mol/L, pH 13) (9:1, v/v). In preliminary trials, this solving buffer did not show any significant antifungal activity (data not shown). Solutions of quercetin, scopoletin or scoparone at concentration 1 mg/mL were obtained by appropriate dilutions of the respective stock solutions.

### 3.3. Penicillium Conidial Suspension

To produce inoculum, a strain of *P. digitatum* molecularly and morphologically identified and deposited in the “Fungal Culture Collection” of the Department of Soil, Plant and Food Sciences (University of Bari Aldo Moro, Bari, Italy) was cultured on PDA dishes for 8 days at 24 ± 1 °C. The surface of the colony was washed with 6 mL of sterile distilled water containing 0.05% (v/v) Tween 80. The resulting suspension was filtered through two layers of sterile gauze and spores were counted by a Thoma chamber (HGB Henneberg-Sander GmbH, Lutzellinden, Germany). A suspension with a concentration of 5 × 10^4^ conidia/mL was used for all *in vitro* and *in vivo* trials.

### 3.4. In Vitro Trials

Aliquots of quercetin, scopoletin and scoparone stock solutions (10 and 1 mg/mL) were incorporated into molten PDA before pouring into Petri dishes (90 mm diameter, 10 mL/dish) so to reach 100 and 10 µg/mL as final concentrations (corresponding to 1000 and 100 µg/dish). Plates were centrally inoculated with 10 µL of *P. digitatum* suspension (500 conidia), and incubated at 24 ± 1 °C in the dark for 6 days. For each compound and concentration, three dishes were prepared. Non amended PDA plates were used as a control. Colony growth (average of the two orthogonal diameters) was recorded at 3 and 6 days post-inoculation (DPI). The experiment was performed twice.

### 3.5. In Vivo Trials

Forty-eight oranges cv. Navelina were surface-sterilised with Na-hypochlorite (2 min in a 2% solution), rinsed under running tap water for 1 min and allowed to dry before wounding with a sterile nail (3 × 3 mm). Then, 10 µL of phenolic buffer solution (10 mg/mL, 100 µg/wound) were pipetted into each wound and, after drying (approximately 30 min later), wounds were inoculated with 10 µL of pathogen conidial suspension (500 conidia). Wounds treated with solving buffer served as a control. Each treatment was replicated three times and each replicate consisted of a tray containing four oranges with three wounds each. Replicates were individually wrapped into a plastic bag, avoiding contact with wounds, and incubated at 24 ± 1 °C for 14 days. Disease incidence (infected wounds, %) and severity (lesion diameter, mm) were recorded every 2 days for 14 days. The experiment was performed twice.

### 3.6. Statistical Analysis

Data were subjected to ANOVA (one-way analysis of variance). Significant differences (*p* ≤ 0.05) were tested by the General Linear Model (GLM) procedure using the Duncan’s Multiple Range Test (DMRT) for colony growth and the Fisher’s Least Significant Difference (LSD) for disease severity and incidence. Percentage data were arcsine-square-root transformed before ANOVA analysis. Data were processed using the statistical software package Statistics for Windows (StatSoft, Tulsa, OK, USA).

### 3.7. Control Index Calculation

The effect of phenolics was expressed by a control index [CI] calculated with the following formula:

CI (%) = [(A − B)/A] × 100
(1)

where A and B correspond to the mean colony diameter or percentage of infected wounds or lesion diameter measured in control (not amended) dishes/oranges and treated dishes/oranges, respectively.

## 4. Conclusions

In conclusion, quercetin, scopoletin and scoparone proved to be effective in reducing green mould severity and incidence in “Navelina” oranges. Since we recorded no consistent effect on fungal growth, further trials are in progress to confirm their activity on a larger scale and to elucidate their possible mode(s) of action.
